# Global differences in the prevalence of the CpG island methylator phenotype of colorectal cancer

**DOI:** 10.1186/s12885-019-6144-9

**Published:** 2019-10-17

**Authors:** Shailesh Mahesh Advani, Pragati Shailesh Advani, Derek W. Brown, Stacia M. DeSantis, Krittiya Korphaisarn, Helena M. VonVille, Jan Bressler, David S. Lopez, Jennifer S. Davis, Carrie R. Daniel, Amir Mehrvarz Sarshekeh, Dejana Braithwaite, Michael D. Swartz, Scott Kopetz

**Affiliations:** 1 Department of Gastrointestinal Medical Oncology, The University of Texas MD Anderson Cancer Center, 1515 Holcombe Blvd, Unit 0426, Houston, TX 77030 USA; 2 Cancer Prevention and Control Program, Lombardi Comprehensive Cancer Center, Georgetown University, Washington DC, 20007 USA; 3 Social Behavioral Research Branch, National Human Genome Research Institute, National Institute of Health, Bethesda, MD 20892 USA; 4 Radiation Epidemiology Branch, Division of Cancer Epidemiology and Genetics, National Institutes of Health, National Cancer Institute, Rockville, MD 20850 USA; 5 Department of Biostatistics and Data Science, School of Public Health, The University of Texas Health Science Center at Houston, Houston, TX 77030 USA; 6 Library, School of Public Health, The University of Texas Health Science Center at Houston, Houston, TX 77030 USA; 7 Department of Epidemiology, Human Genetics and Environmental Sciences, School of Public Health, The University of Texas Health Science Center at Houston, Houston, TX 77030 USA; 8 Division of Urology- UTHealth McGovern Medical School, Houston, TX 77030 USA; 9 Department of Preventive Medicine and Community Health, UTMB Health-School of Medicine, Galveston, TX 77555-1153 USA; 10 Department of Epidemiology, The University of Texas MD Anderson Cancer Center, Houston, TX 77030 USA

**Keywords:** Epigenetics, Methylation, Geographic, CIMP, Colorectal

## Abstract

**Background:**

CpG Island Methylator Phenotype (CIMP) is an epigenetic phenotype in CRC characterized by hypermethylation of CpG islands in promoter regions of tumor suppressor genes, leading to their transcriptional silencing and loss of function. While the prevalence of CRC differs across geographical regions, no studies have compared prevalence of CIMP-High phenotype across regions. The purpose of this project was to compare the prevalence of CIMP across geographical regions after adjusting for variations in methodologies to measure CIMP in a meta-analysis.

**Methods:**

We searched PubMed, Medline, and Embase for articles focusing on CIMP published from 2000 to 2018. Two reviewers independently identified 111 articles to be included in final meta-analysis. We classified methods used to quantify CIMP into 4 categories: a) Classical (MINT marker) Panel group b) Weisenberg-Ogino (W-O) group c) Human Methylation Arrays group and d) Miscellaneous group. We compared the prevalence of CIMP across geographical regions after correcting for methodological variations using meta-regression techniques.

**Results:**

The pooled prevalence of CIMP-High across all studies was 22% (95% confidence interval:21–24%; I^2^ = 94.75%). Pooled prevalence of CIMP-H across Asia, Australia, Europe, North America and South America was 22, 21, 21, 27 and 25%, respectively. Meta-regression analysis identified no significant differences in the prevalence of CIMP-H across geographical regions after correction for methodological variations. In exploratory analysis, we observed variations in CIMP-H prevalence across countries.

**Conclusion:**

Although no differences were found for CIMP-H prevalence across countries, further studies are needed to compare the influence of demographic, lifestyle and environmental factors in relation to the prevalence of CIMP across geographical regions.

## Background

Colorectal cancer (CRC) poses a significant public health burden globally. In 2018, globally, CRC will account for approximately 6.1% of all new cancer cases (*n* = 1,096,601) and 5.8% of cancer related deaths (*n* = 551,269) globally [1]. Disparities in the incidence of CRC occur across geographical regions, with the highest incidence reported in Australia and lowest incidence in Africa [2]. Additionally, mortality rates associated with CRC vary across the globe, with high rates of mortality associated with CRC across Europe as compared to low mortality rates in Africa [2]. Furthermore, differences in CRC incidence exists within regions across age groups, gender, and racial/ethnic groups due to multiple factors, including lifestyle, screening behaviors, and biological factors [3]. Hence, understanding the prevalence and distribution of CRC could help guide tailored prevention, screening and treatment strategies.

The biological processes underlying the development of CRC are complex, with multiple genetic and epigenetic alterations dictating the transition from a normal colon epithelium to cancer. In recent years, much attention has been paid to the role of epigenetic alterations—changes in phenotype or gene expression that do not involve DNA sequence changes—in the development of CRC [4, 5]. One type of epigenetic alteration associated with cancer is methylation of CpG islands in promoter regions of tumor suppressor genes which form hotspots for methylation changes. The CpG island methylator phenotype was first described in CRC by Toyota et al. who identified cancer specific methylation of tumor markers in CRC tissues and is now considered to be an initiating event of the serrated adenoma pathway in the development of CRC [6, 7]. CIMP -High (CIMP-H) is now considered a distinct molecular subtype of sporadic colorectal cancer. It is characterized by a high degree of methylation in promoter-associated CpG-rich regions of tumor suppressor genes, which causes transcriptional inactivation of these genes and leads to cancer development and progression [4, 8].

Large-scale geographic comparisons of CIMP prevalence are limited because there is no consensus on the definition of CIMP and a wide array of methods used for measuring it [8]. In a recent systematic review, Hughes et al. concluded that no gold standard exists for determining CIMP and that no methodology has been shown to be superior to another [9]. Additionally, Jia et al. showed that the estimated prevalence of CIMP-H in CRC tumors ranged from 7 to 48%, and described 16 different of varied genetic markers used to measure CIMP that were based on different threshold values of methylation at individual markers in panels or based on different cut-offs used for defining CIMP-H [10]. The first panel used to measure CIMP, termed the classical panel measures CIMP using the methylated in tumor (MINT) markers *MINT1*, *MINT2, MINT31*, *CDKN2A,* and *MLH1* and defines CIMP-H tumors as those expressing 2 or more of these methylated markers [4, 6]. Subsequently, Weisenberger et al. [11] measured CIMP using a 5-marker panel consisting of *CACNA1G, IGF2, NEUROG1, RUNX3,* and *SOCS1*, and Ogino et al. [12] extended the Weisenberger panel, adding *CDKN2A, CRABP1,* and *MLH1*. These two panels have been widely used to measure CIMP. More recently, genome-wide methylation arrays have been used to study methylation changes across the human genome [13]. Technologies for quantifying CIMP have rapidly developed and include methylation-specific polymerase chain reaction (PCR), real-time PCR (such as MethyLight), and bisulfite pyrosequencing [9]. These technologies measure methylation differently: some at a single gene site, others across several CpG sites of a gene, and others across several genes [9, 14]. Previous reviews have identified significant heterogeneity in pooled estimates of the clinical, pathological, and molecular characteristics of CIMP tumors, particularly due to differences in the methods used to assess CIMP across studies, selection criteria for CRC patient populations, laboratory methods, or geographic region of study [4, 8, 15]. Hence, it is important to consider utilization of CIMP methodologies across geographic areas while studying the geographical variation of estimates of CIMP prevalence.

Lifestyle factors are also shown to be associated with epigenetic changes and might influence prevalence of CIMP phenotype and CRC across geographical regions. Identifying potential differences in CIMP prevalence can help us explore potential factors underlying these differences and explain disparities in the incidence of CRC across geographical regions. It is, therefore, important to understand how CIMP prevalence varies geographically. The aim of this systematic review and meta-analysis was to assess the prevalence of CIMP across geographical regions after correcting for methodological variations using meta-regression techniques.

## Methods

We utilized data from a comprehensive systematic review of clinical, pathological and molecular characteristics of CIMP tumors in CRC [15]. The original systematic review was registered with PROSPERO (registration number CRD42016034181). Included studies had to describe patients who were diagnosed with sporadic CRC and evaluated CIMP phenotype among these patients. We excluded studies that focused on hereditary CRC syndromes and studies focusing on premalignant lesions such as adenomas or polyps within CRC. Additionally, studies focused on other cancers or that did not have a clear description of the measurement or quantification of CIMP were excluded. Original research published in the journal literature was required; review articles were excluded as were conference proceedings. Lastly, we limited articles to those published in English, in peer-reviewed journals.

### Search strategy

The Medline (Ovid), PubMed (National Library of Medicine), and Embase (Ovid) databases were searched with the assistance of a health sciences librarian (HV). The initial searches were completed in April 2016; two updated PubMed searches were completed, one on January 3, 2017 and the other on April 25, 2018. The three main concepts that made up our search were: CIMP or CpG island methylator phenotype/methylation, sporadic CRC, and clinical/pathological and molecular characteristics. A combination of medical subject heading (MeSH) terms and terms included in the title, abstract, and keywords were used to develop the initial Medline search. This search was then adapted for the other databases. Additional file 1 provides an overview of the search strategies used for each database. RefWorks (ProQuest) was used to store all citations found in the search process and to check for duplicates.

### Study selection

An online random number generator (https://www.random.org/integers/) was used to create a random sample of 66 numbers that were then input into an Excel workbook designed specifically for the Cohen’s kappa interrater reliability test [16]. If there were any duplicate numbers, one was replaced by choosing a number between the pair and the number below or above. The numbers corresponded to line numbers within the Excel workbook which resulted in a random sample of titles and abstracts; authors and journal titles were not included in the sample. Two authors (SA, PA) independently screened the sample and reached good interrater agreement (Cohen’s κ = 0.77). After resolving discrepancies in the interrater reliability test through mutual discussion, the same authors then independently screened all titles and abstracts, still blinded to authors and journal titles, using an Excel workbook designed specifically for this step of the systematic review process [16]. Data were compiled into a single Excel workbook at which time consensus was reached on items in which there was disagreement. Articles considered for inclusion were independently reviewed by two authors (SA, PA) and consensus reached by discussion on any disagreements for inclusion. The Excel workbooks also served as our primary tool for gathering all search strategy data and for the creation of the Preferred Reporting Items for Systematic Reviews and Meta-Analyses (PRISMA) flowchart [17].

### Data abstraction

The primary author (SA) identified key study characteristics, including study design, first author, cohort description, and country, and extracted information on the panel markers and/or methodology used to determine CIMP, the cutoff for classifying various CIMP groups in each study, and the prevalence of each CIMP subgroup.

### CIMP classification

Studies generally classified CIMP into three groups: CIMP-High, CIMP-Low and CIMP-0. A few studies also classify CIMP as CIMP+ and CIMP-. For analytical purposes, we coded the classifications of CIMP-H and CIMP+ as CIMP-H and analogously, we coded CIMP-Low, CIMP-, and CIMP-0 as CIMP-0.

### Methodological classification

We classified CIMP methodologies into four groups: (i) the classical panel or MINT marker group, (ii) Wiesenberger-Ogino (W-O) marker panels, and (iii) human methylation array panels and (iv) Miscellaneous. The human methylation arrays included the Infinium HumanMethylation27 BeadChip and Infinium HumanMethylation450 BeadChip (Illumina, Inc., San Diego, CA) [18, 19]. Methodologies that did not fit into any of these 3 categories were categorized as “miscellaneous.”

### Meta-analysis

We calculated the pooled prevalence of CIMP-H across studies using a random effects model [20]. A measure of study heterogeneity (I^2^) was calculated; (I^2^) of > 50% is considered to indicate statistical heterogeneity. Using this method, we calculated pooled prevalence estimates of the CIMP-H subcategory for Asia, Australia, Europe, North America, and South America. No pooled estimate could be calculated across Africa because only one study was identified from the African continent. Additionally, we calculated the pooled prevalence of CIMP-H phenotype across methods described above. Furthermore, country specific prevalence of the CIMP-H group was estimated using a random effects model.

### Meta-regression

We performed a meta-regression analysis to compare CIMP-H prevalence across methodological groups and geographical regions using a random effects model [21]. In the first step, using unadjusted meta-regression analysis, we compared the mean prevalence of CIMP-H across continents, using North America as the reference group. To assess whether differences in methodology accounted for differences in CIMP-H prevalence across continents, we reran meta-regression analysis across geographical regions adjusting for methodological subtype.

### Bias and quality assessment

We performed quality assessment on the included studies. For cohort and case-control studies, the Newcastle-Ottawa Scale was utilized [22]. This scale assesses quality of included studies on three groups: Selection, Comparability, and Assessment. For cohort studies, these include selection strategy of cohort, comparability of exposed and non-exposed cohort, and assessment of outcome and follow-up data. For case control, these include selection strategy of cases and controls, comparability of cases and controls, and ascertainment of exposure, including ascertainment of cases and controls and response rates. Reviewers rate studies on scale of 0–4 for selection, scale of 0–2 for comparability, and scale of 0–3 for ascertainment respectively. Egger’s test was utilized to assess for publication bias due to sample size [23]. Finally funnel plots were plotted to assess for publication bias [24].

## Results

### Screening process

**Fig. 1:**
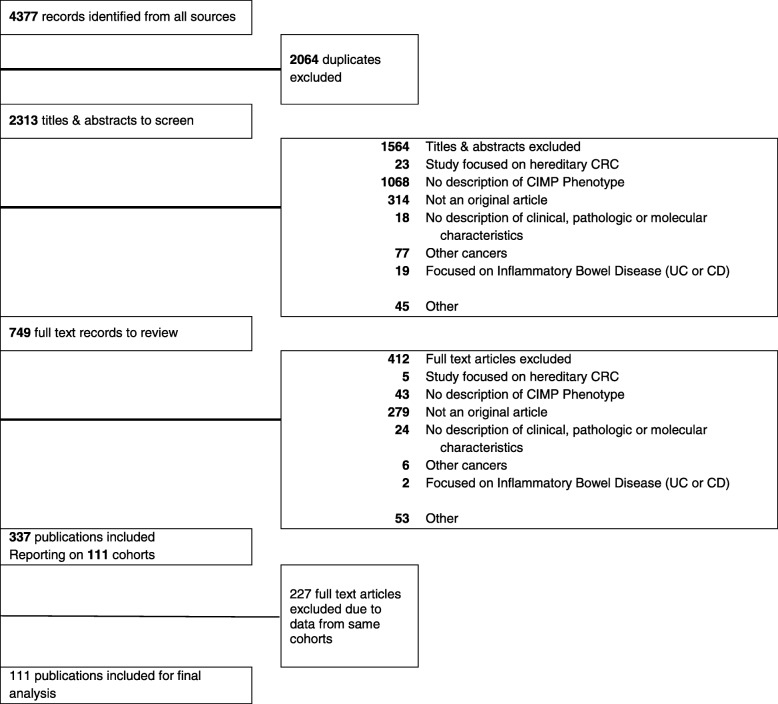
PRISMA flowchart

Our search identified 4377 records. After removal of duplicates, two screeners (SA, PA) screened 2313 abstracts and identified 749 articles for full text review. Figure 1 provides a PRISMA flowchart of the screening process. Of total 749 screened, 279 articles were excluded because they did not report original research, 43 articles did not include a clear description of CIMP, and 90 articles were excluded for other reasons, including a focus on premalignant lesions such as adenomas or polyps or inadequate information on CIMP measurements or methodologies. The final sample consisted of 337 publications for overall analysis.

### Patient population

The 337 included articles represented a total of 111 cohorts and 26 countries. To avoid bias from repeated inclusion of the same patients, we restricted our analysis to 111 cohorts, using data from each cohort with largest sample size. Our final analysis was based on 111 studies that included 37,585 CRC patients (Fig. 1).

### Meta-analysis of CIMP prevalence

We calculated a pooled prevalence of CIMP-H across the included 111 studies [14, 25–133]. The pooled prevalence of CIMP-H was 22% (95% confidence interval [CI], 21–24%; I^2^ = 95.68%). Additionally, 30 studies reported prevalence of CIMP-low (CIMP-L) and CIMP-0. The pooled prevalence of CIMP-L was 34% (95% CI, 30–39%; I^2^ = 94.73%), and the pooled prevalence of CIMP-0 was 47% (95% CI, 40–53%; I^2^ = 96.65%) [25, 27, 30, 35, 41, 44–46, 49, 50, 52, 54, 57, 59, 60, 62, 64, 76, 77, 83, 105, 111, 117–119, 121, 123, 126, 131, 133].

### Summary of CIMP methodologies

The included studies provided information on over 60 different methods or combinations of markers/panels to determine or measure CIMP (Additional file 2: Table S2). We classified these methodologies into 4 groups: MINT marker panel (classical panel), W-O panels, and the human methylation arrays (27 k or 450 k panel). Methods that did not fit into either three were grouped as “miscellaneous”. Additional file 2: Table S2 provides a table with detailed information on the methods used in the studies, including the cutoffs used to define CIMP subgroups and the countries these methods were used in.

### Measuring CIMP-H prevalence across continents

**Fig. 2:**
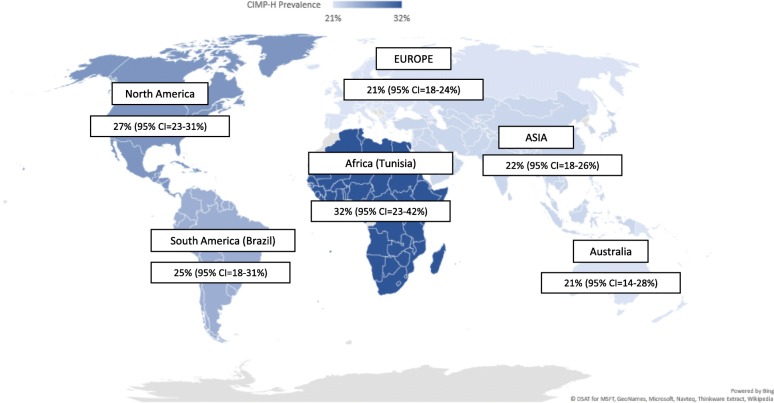
CIMP-H prevalence across continents. Global Map Showing Pooled CIMP-H prevalence across continents. Estimates included pooled prevalence with 95% Confidence Intervals

We calculated pooled prevalence estimates of CIMP-H for Asia, Europe, Australia, North America and South America using random effects model. The pooled CIMP-H prevalence across Asia, Australia, Europe, North America and South America were 22% (95% CI, 18–26%; I^2^ = 95.74%); 21% (95% CI, 14–28%; I^2^ = 97.77%); 21% (95% CI, 18–24%; I^2^ = 94.78%); 27% (95% CI, 23–31%; I^2^ = 96.76%) and 25% (95% CI, 18–31%; I^2^ = not available), respectively. Only 1 study was available from the African subcontinent, from Tunisia. Nine studies included more than 1 country and were not included in the analysis of prevalence across continents [25, 42, 44, 45, 63, 69, 88, 108, 115]. Figure 2 provides an overview of pooled prevalence across countries.

### CIMP prevalence by methodology

We measured the pooled prevalence of CIMP-H for each of the 3 methods used to determine CIMP: MINT marker panel, W-O panel, and human methylation arrays. The pooled prevalence of CIMP-H across these groups were 26% (95% CI, 23–29%; I^2^ = 93.89%); 21% (95% CI: 18–23%; I^2^ = 96.70%) and 22% (95% CI: 13–31%; I^2^ = 91.06%), respectively.

### Meta-regression analysis

Both unadjusted and adjusted meta-regression analysis identified no significant differences in CIMP-H prevalence across geographical regions (Table 1).

**Table 1:** Metaregression analysis of comparison of CIMP-H prevalence across continents. Using North America as the reference group, pooled prevalence using unadjusted and adjusted estimates are shown

	N Studies	N (Total sample size)	N (Total CIMP-H)	Mean CIMP-H prevalence	95% confidence interval	*P*-Value for unadjusted metaregression	*P*-Value for adjusted metaregression
North America	19	13,138	2162	27%	23–31%	Ref	Ref
Asia	30	6628	1113	22%	18–26%	0.24	0.35
Australia	12	4315	618	21%	14–28%	0.19	0.35
Europe	37	7926	1583	21%	18–24%	0.15	0.31
South America	2	159	40	25%	18–31%	0.66	0.90

### Prevalence by country

**Fig. 3:**
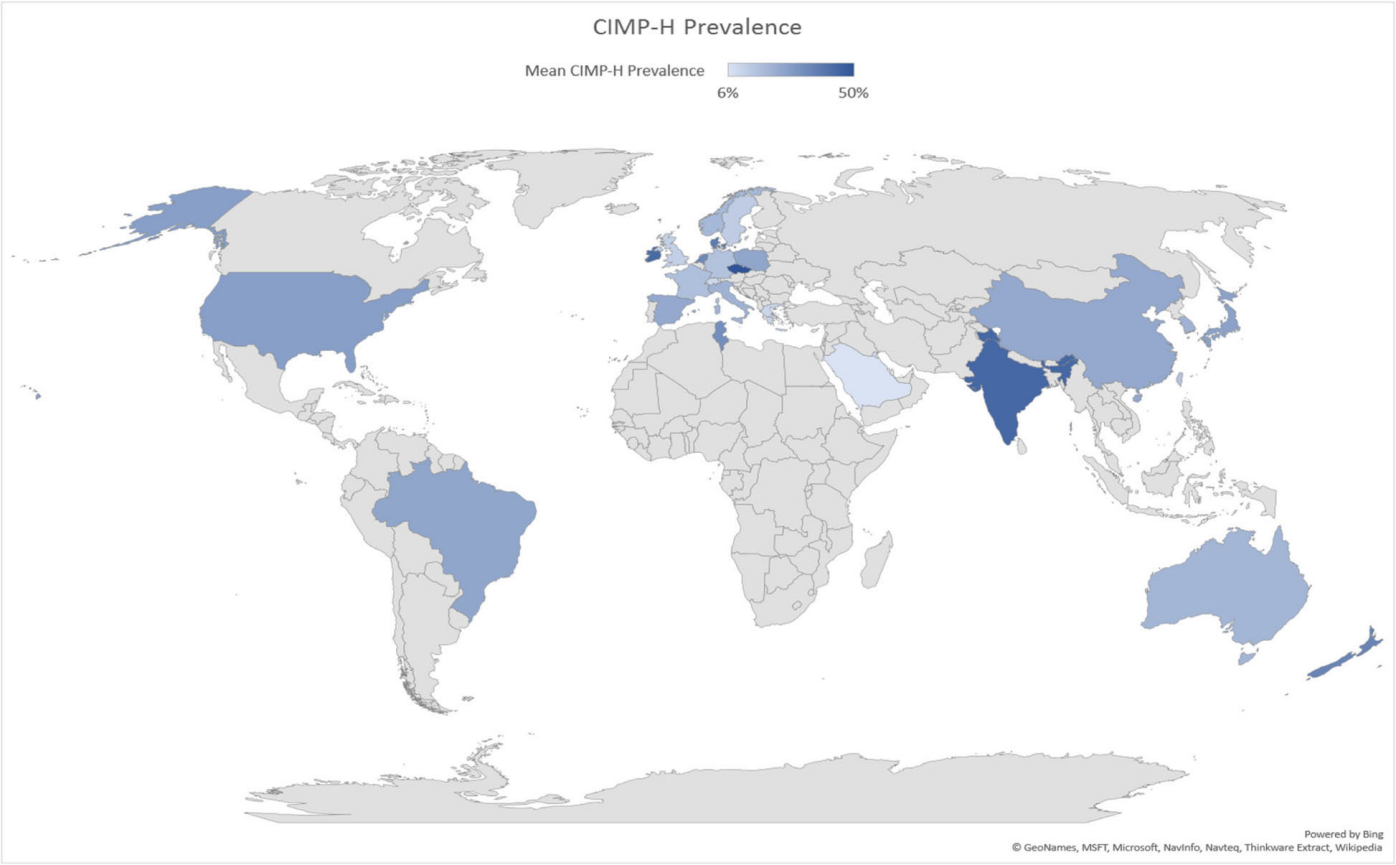
CIMP-H prevalence across countries

Based on available data, we calculated CIMP-H prevalence across countries, and found that CIMP-H prevalence varied widely across countries globally, and within continents (Table 2). CIMP-H prevalence ranged from a low of 6% in Saudi Arabia to high of 50% in Czech Republic. Figure 3 provided a global representation of variation in CIMP-H prevalence across countries.

**Table 2:** Meta- Analysis for CIMP-H prevalence across countries

Continent	Country	Mean CIMP-H prevalence	95% CI	Smoking %	Alcohol drinkers %	Obesity%
Africa	Tunisia	32%	23–42%	40.93%	20%	27%
Australia	Australia	20%	13–27%	14.8%	11.9%	29%
	New Zealand	35%	26–45%	14.9%	12%	31%
Asia	China	24%	12–35%	25.2%	10.6%	6%
	India	44%	37–51%	11.3%	22.3%	4%
	Japan	26%	20–32%	22.5%	9.90%	4%
	Taiwan	16%	12–20%	18%	NA	NA
	South Korea	21%	11–31%	23.6	19.90%	7%
	Kuwait	9%	5–16%	19.9%	3.10%	38%
	Saudi Arabia	6%	3–10%	13.6%	NA	35%
Europe	Greece	10%	6–15%	43.7%	15%	25%
	Italy	21%	14–28%	23.8%	13%	20%
	Spain	24%	18–31%	29.4%	21%	24%
	France	17%	15–19%	32.9%	14.90%	22%
	Switzerland	9%	6–12%	25.8%	13.40%	20%
	United Kingdom	12%	1–24%	22.4%	15.60%	28%
	Netherlands	34%	21–48%	25.9%	13.90%	20%
	Norway	19%	17–21%	20.2%	8.70%	23%
	Sweden	13%	11–15%	18.9%	12.50%	21%
	Denmark	38%	35–42%	19.1%	14.40%	20%
	Germany	16%	12–20%	30.7%	13.40%	22%
	Poland	25%	19–31%	28.2%	17.90%	23%
	Czech Republic	50%	42–59%	34.4%	19.50%	26%
	Ireland	43%	29–58%	24.4%	19.30%	25%
North America	United States	27%	23–31%	21.9%	14.40%	36%
South America	Brazil	25%	18–31%	14%	18.50%	22%

*CIMP-H* CIMP-High, *CI* 95% Confidence Interval, *%* Prevalence as % using World Health Organization data, *NA* Not available

### Correlation of CIMP-H phenotype with prevalence of lifestyle factors across countries

Using statistics from the World Health Organization [134], we measured the prevalence of risk factors previously associated with CRC [135–137]: obesity (BMI > =30 kg/m^2^), alcohol intake (ever drinkers) and smoking (ever-smoker) with measured CIMP-H prevalence across countries (Table 2). CIMP-H was significantly corelated with alcohol intake (Pearson correlation = 0.51, *p* = 0.01). No significant correlation was observed with the prevalence of obesity (Pearson correlation = − 0.24, *p* = 0.23) or smoking (Pearson correlation = − 0.004, *p* = 0.98). Though these correlations do not imply causation, it provides evidence of possible association of alcohol intake associated epigenetic changes with CIMP phenotype in CRC and warrants further exploration in future studies.

### Bias and quality assessment

Our results identified significant heterogeneity as assessed by funnel plots for pooled prevalence across studies. Additional file 3: Figure S1 depicts funnel plots for assessing publication bias for pooled CIMP-H prevalence. Additionally, possible heterogeneity was also observed in funnel plots for CIMP-H prevalence measures across panel types and continents (Additional file 3). We found evidence for publication bias, as seen by significant Egger’s Publication Bias test (*p* = 0.014) in pooled CIMP-H prevalence, as well as in pooled prevalence across North America (*p* = 0.03). Quality assessment results for cohort and case control studies using the New-Castle Ottawa scale has been summarized in Additional files 4 and 5 respectively.

## Discussion

To our knowledge, this is the first systematic review to assess differences in the prevalence of CIMP phenotype in CRC across geographical regions. We found pooled prevalence of CIMP-H to be 22% across all studies. Additionally, the pooled prevalence of CIMP-H varied from 21 to 27% across geographical regions, and meta-regression analysis did not identify any significant differences in pooled prevalence across geographical regions, after adjusting for methodological differences. Moreover, our literature search identified over 60 combinations of panels and methods to measure CIMP, highlighting the rapidly evolving methodologies and technologies used to measure CIMP, differences in its utility across geographical regions, and the need to develop consensus on identifying a gold standard for measuring CIMP across geographical regions.

The incidence of CRC varies across different regions of the world [138, 139]. For example, the overall incidence of CRC in Australia is among the highest in the world, approximately 40% higher than in the United States (12% vs 8.6%) [139]. Previous studies have also found a higher incidence of CRC and a higher ratio of colon to rectal cancers in Western than in Asian populations [138–141]. The reasons for these differences are likely complex and involve differences in risk factors associated with CRC including lifestyle, genetic and epigenetic factors as well as screening practices across geographical regions [142]. Though our analysis did not find significant differences in CIMP prevalence across geographical regions, variation in other molecular subsets across geographical regions is warranted. CRC has been shown to be associated with lifestyle factors such as a diet high in red or processed meat, inadequate consumption of fruits and vegetables, smoking, obesity, and lack of physical activity [143]. Lifestyle factors have been postulated to induce inflammation through various mechanisms including epigenetic changes like hypermethylation [144]. Alcohol consumption may influence carcinogenesis through several possible mechanisms, including altering DNA methylation patterns by affecting intestinal absorption, hepatobiliary metabolism, and renal excretion of folate [145]. One study in Germany found higher consumption of alcohol among patients diagnosed with CIMP-L (but not CIMP-H) CRC tumors [110]. Another study found that the type of alcohol consumed (wine or beer) can affect the development of CIMP tumors [146]. Smoking too has been shown to be associated more with proximal than distal CRCs, suggesting differential changes induced by tobacco exposure on CRC pathways [147]. As CIMP is more common in proximal than in distal CRCs, smoking may be involved in the development of CIMP-high cancers associated with proximal tumor location [11]. Samowitz et al. reported higher odds of CIMP-H CRC tumors among smokers versus nonsmokers (OR = 2.06, 95% CI = 1.43 to 2.97) [148]. In a study comparing the development of CIMP tumors by smoking status, a shorter time since cessation of smoking was associated with a higher risk of developing CIMP-H CRC [149]. Slattery et al. observed an association between a high body mass index (BMI) and CIMP- Low but not CIMP-High colon tumors and no association between BMI and CIMP status in rectal tumors [150]. In addition, one study reported that overweight or obesity was associated with the development of CIMP-H tumors [29].

Dietary factors also play a key role in CRC development. Certain dietary components have shown to be associated with inducing systemic and gastrointestinal specific inflammation, leading to increase in levels of pro-inflammatory cytokines including IL-1, IL-6 and TNF-a, and activation of downstream oncogenic signaling pathways [151]. Differences in total and red meat intake have been observed between the United States and other developed countries [152]. Diets low in methyl-contributing folate, vitamins B6 and B12, methionine and high consumption of alcohol, affect DNA methylation and have been shown to result in increased risk of cancers with CpG island methylation [145]. Lastly, the combination of high inflammatory diets comprising of saturated fatty acids, high levels of sugar, red and processed meat, coupled with low dietary fibers and green leafy vegetables promote inflammation through increase in levels of pro-inflammatory cytokines [151]. In normal healthy colon tissue from women, obesity and smoking increased DNA methylation at genes hypermethylated in cancer, but aspirin and hormone replacement therapy reduced DNA hypermethylation [153]. Therefore, a better understanding of differences in the prevalence of risk factors among CIMP subgroups across geographical regions could help unravel the pathways involved in CIMP CRC and explain disparities in CIMP prevalence across these regions. Racial, ethnic, and genetic differences in CIMP prevalence have also been previously reported. For instance, compared to patients of Southern European origin, the prevalence of CIMP tumors was higher in Australian-born CRC patients of Anglo-Celtic origin, suggesting a genetic predisposition to CIMP tumors among the latter population [154]. Other studies have also found evidence for a genetic basis for CIMP; for example, polymorphisms in genes such as *MSH6 and MTHFR* allele have been shown to be associated with CIMP-H tumors across diverse populations [151, 152, 155]. In addition, the authors also observed a joint effect of low folate, low methionine, high alcohol consumption, and *MTHFR* 1298 AC or CC genotypes on the risk of CIMP+ CRC in the US study [156]. Hence possible differences in genetic makeup across countries can also underline differences in CIMP-H prevalence across geographical regions. Future studies should focus on a comprehensive assessment of lifestyle and genetic factors to compare differences and incidence of CIMP+ cancers across geographical regions.

Differences in utility of panels/methods for measuring CIMP significantly differed across continents, highlighting the rapid pace of development of the understanding of role of epigenetics in cancer and the associated technology and methodology used to quantify understanding of this phenotype. CIMP was first characterized by Issa et.al as a subset of highly methylated CRCs with features of concordant hypermethylation of multiple CpG island loci and bimodal distribution of the number of methylated CpG island loci using the 7-marker MINT panel [157]. The classical MINT marker panel paved the way for understanding methylation changes at the gene level. Lee et al. [158] later compared CIMP tumors using the classical and the Weisenberger panels. The Weissenberg panel was better at identifying the clinical, pathological, and molecular characteristics of CIMP tumors and worse at prognosis [158]. In 2007, Ogino et al. [12] proposed that a panel of 4 markers for CIMP—*RUNX3*, *CACNA1G*, *IGF2*, and *MLH1*—should be sensitive and specific enough for research and clinical use. Recently, the use of human methylation arrays have opened the door to an understanding of methylation across the whole genome [19]. Another difference among studies of CIMP is the utilization of primers and probes as well as identification of “CpG” probes and their location on the gene [9]. Differences in these methodological factors, in addition to differences in the background populations and thus the cohort compositions, might explain some of the variation in results obtained using different CIMP panels [76]. Other factors may include methods of tissue preservation, screening modalities, and patient selection criteria. This highlights the rapid pace of development of field of Epigenetics and transition from gene-specific panels to study of genome wide methylation across cancer patients.

The study of epigenetic modifications like CIMP is of growing interest to the fields of public health and cancer prevention, detection, and management. Assessing differences in the prevalence of CIMP across regions of the world is key to identifying factors that contribute to these changes and to developing primary cancer prevention strategies. CIMP can be present in precancerous “normal” tissue, raising cancer risk [159–162]. Because recent combined genetic and epigenetic analyses of sporadic CRC identified subsets possessing distinct clinicopathologic features, elucidation of the precise roles of epigenetic abnormalities such as CIMP might be a great help for the prevention, screening, and treatment of CRCs [163].

Our study has several strengths. First, we did not restrict our search strategy to any country or group of CRC patients. Second, to maintain uniformity we excluded CIMP studies focusing on precancerous lesions such as adenomas and polyps, as CIMP is considered to evolve from the serrated adenoma pathways [57, 164, 165]. Our study also had some limitations. First, because no gold standard exists for defining CIMP, comparisons of studies across panels or countries are challenging. Also, genetic markers across panels varied, including differences in cut-offs for defining CIMP groups within each methodological subtype too, which might add to possible heterogeneity in pooled prevalence of CIMP-H overall and across regions. Second, the high degree of heterogeneity in the results highlights the biological complexity of CIMP. Third, a few studies utilizing Weisenberger or Ogino panel had different cut-offs to determine CIMP within the same methodology which might lead to difference in prevalence of CIMP-H. Also the sensitivity of different methodologies or gene panels to quantify methylation might vary, possibly adding another source of heterogeneity in our results. Lastly, meta-regression has low power, and controlling for multiple factors in meta-regression analysis is not recommended. Hence, we decided to control only for methodology. By limiting to reports published in English, we may have excluded entire regions which report their findings in other languages. Though we identified positive correlation of CIMP-H prevalence with alcohol intake, these correlations do not imply causation and warrants further exploration in future studies. Finally, our results should be interpreted with caution due to small sample size in subgroup analysis and high heterogeneity identified in pooled analysis which might be due to factors other than methodology including differences in population characteristics, sampling variation, or by chance alone.

Through our study, we highlight the importance of considering variations in molecular methodologies in examining global variations of a disease phenotype. Further using principles of molecular pathology epidemiology, it is possible to examine the role of lifestyle, demographic and molecular factors in understanding possible mechanisms that explain possible differences in CIMP prevalence across geographical regions.

## Conclusion

In summary, our results identified mean CIMP-H prevalence to be 22%, with no differences observed between geographical regions. However, there were variations observed across countries within continents. In ecological analysis, CIMP-H prevalence showed strong correlation with alcohol intake at a global level, highlighting possible role of epigenetic mechanisms underlying this association. Finally, we also identified variations in methodologies used to quantify CIMP and need to identify a gold standard for making effective comparisons.

## Supplementary information

**Additional file 1.** Search Strategies for Systematic Review and Meta-analysis.

**Additional file 2.** Table summarizing CIMP methodologies.

**Additional file 3.** Funnel and Forest Plots for Meta-analysis.

**Additional file 4.** Bias Assessment for Cohort Studies.

**Additional file 5.** Bias Assessment for Case-Control Studies.

## Data Availability

All materials have been cited in the manuscript.

## Abbreviations

CIMP: CpG island methylator phenotype
CRC: Colorectal cancer
MINT: Methylated in tumor
W-O: Weisenberger –Ogino
