# Supplementary material for: Global differences in the prevalence of the CpG island methylator phenotype of colorectal cancer
Source: BMC Cancer. 2019 Oct 17;19:964. doi: 10.1186/s12885-019-6144-9 (PMC6796359; doi:10.1186/s12885-019-6144-9)
Supplement: Supplementary file 1 — Additional file 1. Search Strategies for Systematic Review and Meta-analysis. [file 12885_2019_6144_MOESM1_ESM.docx]

**Search strategies and results:**

**Table S1: Summary of Databases Searched**

| **Table** | **Vendor/ Interface** | **Database** | **Date searched** | **Database update** | **Searcher(s)** |
| --- | --- | --- | --- | --- | --- |
| 1a | Ovid | Medline^®^ | 4/14/2016 | without Revisions 1996 to April Week 1 2016; In-Process & Other Non-Indexed Citations April 13, 2016 | Helena M. VonVille; Shailesh Advani |
| 1b | National Library of Medicine | PubMed | 4/14/2016; updated 1/3/2017; updated 4/25/2018 | 4/14/2016; updated 1/3/2017; updated 4/25/2018 | Helena M. VonVille; Shailesh Advani |
| 1c | Ovid | EMBASE^®^ | 4/15/2016 | 1996 to 2016 Week 15 | Helena M. VonVille; Shailesh Advani |

**Table S1a: Ovid Medline^®^ search strategy**

| **Provider/Interface** | Ovid |
| --- | --- |
| **Database** | Medline^®^ |
| **Date searched** | 4/14/2016 |
| **Database update** | without Revisions 1996 to April Week 1 2016; In-Process & Other Non-Indexed Citations April 13, 2016 |
| **Search developer(s)** | Helena M. VonVille; Shailesh Advani |
| **Limit to English** | Yes |
| **Date Range** | 1999-2016 |

| **Line #** | **Search statement** | **# found** |
| --- | --- | --- |
| 1 | Colorectal Neoplasms/ | 1787648 |
| 2 | (colon cancer or colonic cancer or colon cancers or colonic cancers or colonic neoplasms or colorectal cancer or colorectal neoplasms or colorectal carcinoma* or rectal cancer or rectal neoplasms).ti,ab,kw. | 287882 |
| 3 | Colonic Neoplasms/ or Rectal Neoplasms/ | 1858396 |
| 4 | 1 or 2 or 3 | 53262 |
| 5 | Methylation/ or DNA Methylation/ | 9291 |
| 6 | (hypermethylation or methylation or hypermethylator or methylator).ti,ab,kw. | 50102 |
| 7 | 5 or 6 | 63884 |
| 8 | 4 and 7 | 226085 |
| 9 | Methyl-CpG-Binding Protein 2/ or CpG Islands/ | 29548 |
| 10 | (CpG or CIMP or MECP2).ti,ab,kw. | 465338 |
| 11 | 9 or 10 | 528211 |
| 12 | 8 and 11 | 90323 |
| 13 | limit 12 to (english language and yr="1999 - 2016") | 2532242 |

**Table S1b: PubMed search strategy**

| **Provider/Interface** | National Library of Medicine |
| --- | --- |
| **Database** | PubMed |
| **Date searched** | 4/14/2016; updated 1/3/2017 |
| **Database update** | 4/14/2016; updated 1/3/2017 |
| **Search developer(s)** | Helena M. VonVille; Shailesh Advani |
| **Limit to English** | Yes |
| **Date Range** | 1999-2016 |

| 1 | Colorectal Neoplasms[mesh:noexp] |
| --- | --- |
| 2 | (colon cancer[tiab] OR colonic cancer[tiab] OR colon cancers[tiab] OR colonic cancers[tiab] OR colonic neoplasms[tiab] OR colorectal cancer[tiab] OR colorectal neoplasms[tiab] OR colorectal carcinoma*[tiab] OR rectal cancer[tiab]or rectal neoplasms[tiab]) |
| 3 | Colonic Neoplasms[mesh:noexp] OR Rectal Neoplasms[mesh:noexp] |
| 4 | #1 OR #2 OR #3 |
| 5 | Methylation[mesh:noexp] OR DNA Methylation[mesh:noexp] |
| 6 | (hypermethylation[tiab] OR methylation[tiab] OR hypermethylator[tiab] OR methylator[tiab]) |
| 7 | #5 OR #6 |
| 8 | #4 AND #7 |
| 9 | Methyl-CpG-Binding Protein 2[mesh:noexp] OR CpG Islands[mesh:noexp] |
| 10 | (CpG[tiab] OR CIMP[tiab] OR MECP2[tiab]) |
| 11 | #9 OR #10 |
| 12 | #8 AND #11 |
| 13 | #12 AND English[la] AND 1999:2016[dp] |
|  | updated 1/3/2017 |
| 13 | #12 AND 2016:2017[dp] |
|  | updated 4/25/2018 |
| 13 | #12 AND 2017:2018[dp] AND english[la] |

**Table S1c: Ovid EMBASE**^®^ **search strategy**

| **Provider/Interface** | Ovid |
| --- | --- |
| **Database** | EMBASE^®^ |
| **Date searched** | 4/15/2016 |
| **Database update** | 1996 to 2016 Week 15 |
| **Search developer(s)** | Helena M. VonVille; Shailesh Advani |
| **Limit to English** | Yes |
| **Date Range** | 1999-2016 |

| 1 | anus cancer/ or cecum cancer/ or colon adenocarcinoma/ or colon adenoma/ or colon cancer/ or colon carcinoma/ or colon metastasis/ or colon polyp/ or colon tumor/ or colorectal adenoma/ or colorectal cancer/ or colorectal carcinoma/ or colorectal tumor/ or metastatic colorectal cancer/ or sigmoid carcinoma/ |
| --- | --- |
| 2 | (colon cancer or colonic cancer or colon cancers or colonic cancers or colonic neoplasms or colorectal cancer or colorectal neoplasms or colorectal carcinoma* or rectal cancer or rectal neoplasms).ti,ab,kw. |
| 3 | 1 or 2 |
| 4 | methylation/ |
| 5 | dna methylation/ or dna methylation assay/ |
| 6 | methylated DNA protein cysteine methyltransferase/ |
| 7 | (hypermethylation or MECP2 or methylation or hypermethylator or methylator).ti,ab,kw. |
| 8 | 4 or 5 or 6 or 7 |
| 9 | 3 and 8 |
| 10 | cpg island/ |
| 11 | methyl CpG binding protein 2/ |
| 12 | (CpG or CIMP or MECP2).ti,ab,kw. |
| 13 | 10 or 11 or 12 |
| 14 | 9 and 13 |
| 15 | mutation/ or chromosome mutation/ or gene mutation/ or genomic instability/ or germline mutation/ or induced mutation/ or mutagenesis/ or mutation rate/ or somatic hypermutation/ or somatic mutation/ or spontaneous mutation/ |
| 16 | gene mutation/ or allelic imbalance/ or deletion mutant/ or frameshift mutation/ or "gain of function mutation"/ or gene deletion/ or gene disruption/ or gene insertion/ or gene loss/ or indel mutation/ or "loss of function mutation"/ or missense mutation/ or mutator gene/ or nonsense mutation/ or null allele/ or point mutation/ or splicing defect/ |
| 17 | molecular pathology/ |
| 18 | signal transduction/ |
| 19 | microsatellite instability/ |
| 20 | chromosomal instability/ or genomic instability/ |
| 21 | Phenotype/ |
| 22 | genetic regulation/ or "chromatin assembly and disassembly"/ or crispr cas system/ or epigenetic repression/ or epigenetics/ or epistasis/ or gene cassette/ or gene control/ or gene expression regulation/ or gene regulatory network/ or gene silencing/ or genetic epigenesis/ or histone modification/ or mating type/ or posttranscriptional gene silencing/ or receptor down regulation/ or receptor upregulation/ or rna interference/ or transactivation/ or transcription regulation/ |
| 23 | (chromosomal instability or epigenetic* or epigenomic* or metastas* or mutate* or mutation* or microsatellite instability).ti,ab,kw. |
| 24 | 15 or 16 or 17 or 18 or 19 or 20 or 21 or 22 or 23 |
| 25 | 14 and 24 |
| 26 | limit 25 to (english language and yr="1999 - 2016") |
| 27 | 26 not medline.cr. |

**Table S2: Non-database searches yielding new studies**

**Scopus results**

Add any citations that you searched in Scopus in which you found studies not located during the database searches.

**Bibliographies searched**

Note: add the citation of the article in which you FOUND the new item.

**Author names searched**

Add any authors names that you searched in which you found studies not located during the database searches.
