# Supplementary material for: Global differences in the prevalence of the CpG island methylator phenotype of colorectal cancer
Source: BMC Cancer. 2019 Oct 17;19:964. doi: 10.1186/s12885-019-6144-9 (PMC6796359; doi:10.1186/s12885-019-6144-9)
Supplement: Supplementary file 2 — Additional file 2. Table summarizing CIMP methodologies. [file 12885_2019_6144_MOESM2_ESM.docx]

| **Table S2: Summary Table for Methodologies for Quantifying CIMP (1999-2018)** | | | | | | |
| --- | --- | --- | --- | --- | --- | --- |
| **Method** | **No of Markers** | **Markers** | **Definition of CIMP-High** | **Definition of CIMP-Low** | **Definition of CIMP-0** | **Countries Used in** |
| MINT Marker | 3 | CDKN2A, MINT2, MINT31 | ≥ 2/3 | NA | NA | NA |
| MINT Marker | 3 | CDKN2A, MINT2, MDR1 | ≥ 2/3 | 0 to 1/3 | 0 to 1/3 | Australia |
| MINT Marker | 4 | CDKN2A, MLH1, MINT1, MINT31 | ≥ 3/4 | 0 to 2/4 | 0 to 2/4 | Japan, USA |
| MINT Marker | 4 | MINT1, MINT2. MINT31, MINT12 | ≥ 3/4 | 1 to 2/4 | 0/4 | Australia |
| MINT Marker | 5 | CDKN2A, MLH1, MINT1, MINT2, MINT31 | ≥ 2/5 or ≥ 3/5 | 0-2/5 or 1-2/5 | 0-2/5 or 0/5 | India, Norway, Taiwan, USA, Australia, Japan, France, Korea, Hong Kong, |
| MINT Marker | 5 | CDKN2A, MINT1, MINT2, MINT31, MINT12 | ≥ 2/5 or ≥ 3/5 | 0 -1/5 or 0-2/5 | 0 -1/5 or 0-2/5 | Japan and Australia |
| MINT Marker | 5 | CDKN2A, MLH1, MINT1, MINT2, MGMT | ≥ 3/5 | 1 to 2/5 | 0/5 | Germany |
| MINT Marker | 5 | CDKN2A, MLH1, MINT1, MGMT, p14 | ≥ 3/5 | 0-2/5 | 0-2/5 | China |
| MINT Marker | 5 | MINT1, MINT2, MINT31, MINT12, MINT25 | ≥ 3/5 | 0-2/5 | 0-2/5 | Japan |
| MINT Marker | 5 | MLH1, MINT1, MINT2, MINT31, MGMT | ≥ 3/5 | 1 to 2/5 | 0/5 | Germany |
| MINT Marker | 6 | CDKN2A, MLH1, MINT1, MINT2, MINT31, p14 | ≥ 2/6 & ≥ 3/6 | 0 to 1/6 or 0 to 2/6 | 0 to 1/6 or 0 to 2/6 | UK, USA, Korea, Japan |
| MINT Marker | 6 | MINT1, MINT2, MINT31, MINT12, TIMP3, RIZ1 | ≥ 4/6 | 0 to 3/6 | 0 to 3/6 | Netherlands |
| MINT Marker | 6 | CDKN2A, MLH1, MINT1, MINT2, MINT31, MGMT | ≥ 3/6 | 0 to 2/6 | 0 to 2/6 | USA |
| MINT Marker | 6 | CDKN2A, MLH1, MINT1, MINT31, RASSF2, H1C1 | ≥ 3/6 | 0 to 2/6 | 0 to 2/6 | USA |
| MINT Marker | 6 | MINT1, MINT2, MINT31, MINT12, MINT27, p16 | ≥ 3/6 | 0 to 2/6 | 0 to 2/6 | USA |
| MINT Marker | 7 | CDKN2A, MLH1, MINT1, MINT2, MINT31, RASSF2, P14 | ≥ 3/7 | 0 to 2/7 | 0 to 2/7 | Japan |
| MINT Marker | 7 | CDKN2A, MINT1, MINT2, MINT31, MINT12, MINT27, MINT17 | ≥ 3/7 | 0 to 2/7 | 0 to 2/7 | USA |
| MINT Marker | 7 | (p16, MLH1, MINT1, MINT2, MINT12, MINT17, and MINT31 | MLH1+≥ 4/6 | 2 to 4/6, no MLH1 | 0 to 1/7, no MLH1 | USA and Japan |
| MINT Marker | 7 | CDKN2A, MLH1, H1CI, MINT1, MINT31, RASSF2, ID4 | ≥ 3/7 | 0 to 1/7 | 0 to 1/7 | USA |
| MINT Marker | 7 | CDKN2A, MLH1, MINT1, MINT2, MINT31, p14, WNT | ≥ 3/7 | 1 to 2/7 | 0/7 | Korea |
| MINT Marker | 7 | CACNA1G, CDKN2A, MLH1, MINT1, MINT2, MINT31, p14 | ≥ 2/7 | 0 to 1/7 | 0 to 1/7 | Korea |
| MINT Marker | 7 | CDKN2A, MLH1, MINT1, MINT31, RASSF1, HIC1, MGMT | ≥ 3/7 | NA | NA | NA |
| MINT Marker | 7 | CDKN2A, MLH1, MINT1, MINT31, RASSF2, H1CI, ID4 | ≥ 3/7 | 0 to 2/7 | 0 to 2/7 | USA |
| MINT Marker | 7 | RIZ1, HTR6, MAP1B, CACNA1G, IGF2, RUNX3, MINT31 | ≥ 4/7 | 0 to 3/7 | 0 to 3/7 | Italy |
| MINT Marker | 7 | CDKN2A, MLH1, H1C1, MINT1, MINT31, RASSF2, MGMT | ≥ 3/7 | NA | NA | NA |
| MINT Marker | 7 | CDKN2A, MLH1, MINT1, MINT2, MINT31, MDR1, MINT12 | ≥ 3/7 | 0 to 2/7 | 0 to 2/7 | USA |
| MINT Marker | 7 | CDKN2A, RUNX3, MLH1, MINT1. MINT31, MGMT, APC | ≥ 4/7 | 1 to 3/7 | 0/7 | China |
| MINT Marker | 6 | CKDN2A, MLH1, MGMT, CDH13, p14, APC | ≥ 2/6 | 0 to 1/6 | 0 to 1/6 | USA |
| MINT Marker | 10 | CDKN2A, MLH1, MINT1, MINT2, MINT31, RASSF1, DAPK, ECAD, BRAC1, GSTP1 | ≥ 5/10 | 1 to 5/10 | 0/10 | India |
| MINT Marker | 7 | CDKN2A, MLH1, MINT1, MINT2, MINT31, MGMT, APC | ≥ 4/7 | NA | NA | NA |
| MINT Marker | 7 | MINT1, MINT2, MINT31, hMLH1, p16, p14, and WNT5A 9505 | ≥ 3/7 | 0 to 2/7 | 0 to 2/7 | Korea |
| MINT Marker | 14 | hMLH1, p16ink4A, HIC1, RASSF2, MINT1 and MINT31, SFRP1, SFRP2, SFRP4, SFRP5, SLC5A8, TAC1, SST and MGMT | ≥ 9/14 & ≥ 12/14 | 0 to 8/14 & 0 to 11/14 | 0 to 8/14 & 0 to 11/14 | USA |
| W-O | 4 | CACN1G, CDKN2A, IGF2 and RUNX3 | ≥ 3/4 | 0-2/4 | 0-2/4 | Japan |
| W-O | 5 | CACNA1G, IGF2, NEUROG1, CRABP1, MLH1 | ≥ 1-5/5 | 0/5 | 0/5 | Denmark |
| W-O | 5 | CACN1G, IGF2, NEUROG1, RUNX3, SOCS1 | ≥ 3/5 or ≥ 2/5 | 0 to 2/5 or 1-2/5 | 0 to 2/5 or 0/5 | USA, Australia, Japan, UK, France, Germany, Italy, Korea, Netherlands, Poland, Saudi Arabia |
| W-O | 5 | CDKN2A, IGF2, RUNX3, SOCS1, NGN1 | ≥ 3/5 | 1 to 2/5 | 0/5 | Australia, Saudi Arabia |
| W-O | 5 | CACNA1G, CDKN2A, IGF2, RUNX3, MLH1 | ≥ 3/5 | 1 to 2/5 | 0/5 | Japan |
| W-O | 5 | CDKN2A, MLH1, IGF2, BDNF, CALCA | ≥ 3/5 | NA | NA | NA |
| W-O | 5 | CACNA1G, CDKN2A, NEUROG1, CRABP1, MLH1 | ≥ 3/5 and ≥ 4/5 | 0 to 3/5 or 0 to 2/5 or 1 to 3/5 | 0 to 3/5 or 0 to 2/5 or 0/5 | Germany, Japan, Switzerland, USA |
| W-O | 5 | CACNA1G, NEUROG1, RUNX3, SOCS1, MLH1 | ≥ 3/5 | 0-2/5 | 0-2/5 | Spain |
| W-O | 6 | CDKN2A, MLH1, MGMT, THSB1, p14, APC | ≥ 3/6 | 0 to 2/6 | 0 to 2/6 | Japan |
| W-O | 6 | CDKN2A, NEUROG1, RUNX3, SOCS1, CRABP1, MLH1 | ≥ 3/6 | 1 to 2/6 | 0/6 | Greece |
| W-O | 6 | CACN1G, CDKN2A, NEUROG1, RUNX3, SOCS1, mutL Homolog | ≥ 3/6 | 1 to 2/6 | 0/6 | Greece |
| W-O | 6 | CACN1G, CDKN2A, IGF2, RUNX3, MLH1, MGMT | ≥ 3/6 | NA | NA | NA |
| W-O | 6 | CDKN2A, IGF2, NEUROG1, RUNX3, SOCS1, MLH1 | ≥ 3/6 | 0 to 2/6 | 0 to 2/6 | Italy |
| W-O | 7 | CACNA1G, IGF2, NEUROG1, RUNX3, SOCS1, CRABP1, MLH1 | ≥ 5/7 | 1 to 4/7 | 0/7 | USA |
| W-O | 7 | CACNA1G, IGF2, NEUROG1, RUNX3, SOCS1, MHL1, p16 | ≥ 3/7 | 0 to 2/7 | 0 to 2/7 | Tunisia |
| W-O | 7 | CACNA1G, CDKN2A, IGF2, NEUROG1, RUNX3, SOCS1, RASSF2 | ≥ 4/7 | 0 to 3/7 | 0 to 3/7 | France |
| W-O | 7 | CACNA1G, CDKN2A, IGF2, NEUROG1, RUNX3, SOCS1, CRABP1 | ≥ 4/7 | 1 to 3/7 | 0/7 | Kuwait |
| W-O | 8 | CACN1G, CDKN2A, IGF2, NEUROG1, RUNX3, SOCS1, CRABP1, MLH1 | ≥ 6/8 and ≥ 5/8 | 1 to 5/8 or 0-5/8 or 0-4/8 | 0/8 or 0-5/8 or 0 to 4/8 | USA, Saudi Arabia, Korea, Sweden, Spain, Netherlands, Czech Republic, |
| Human Methylation Array | 450 K Panel | Hierarchical Analysis | NA | NA | NA | USA, Multiple |
| Human Methylation Array | TCGA 27k Panel | Hierarchical Analysis | NA | NA | NA | Poland, USA, Spain |
| Miscellaneous | 4 | CDKN2A, MLH1, TIMP3, ARF | ≥ 2/4 | 0 to 1/4 | 0 to 1/4 | Japan |
| Miscellaneous | 4 | CKDN2A, MLH1, TPEF, TIMP3 | ≥ 3/4 | NA | NA | NA |
| Miscellaneous | 5 | MLH1, MGMT, GSTP1, THSB1, APC | ≥ 2/5 | 1 of 5 | 0/5 | Korea |
| Miscellaneous | 5 | CDKN2A, MLH1, DAPK, MGMT, p14 | ≥ 2/5 or ≥ 3/5 | 0 to 1/5 or 0 to 2/5 | 0 to 1/5 or 0 to 2/5 | Brazil |
| Miscellaneous | 7 | ER, SFRP1, MYOD1, SLC16A2, SPOK2, N33, MGMT | ≥ 4/7 | 2 to 3/7 | 0 to 1/7 | USA and Japan |
| Miscellaneous | 7 | MLH1, MSH2, MSH6, MLH3, PMS2, MSH3, MGMT | ≥ 5/7 | 1 to 4/7 | 0/7 | Brazil |
| Miscellaneous | 9 | TMEFF2, VIM, CACNA1G, CDKN2A, NEUROG1, CRABP1, MLH1, ESR1, APC | ≥ 5/9 | 1 to 4/9 | 0/9 | China |
| Miscellaneous | 15 | CACNA1G, CDKN2A, MLH1, CDH4, ID4, MGMT, TIMP3, TSP1, HCAD, GATA5, RSASF1A, HTLF, HRK,SLC13, TSLC1 | ≥ 7/15 | 1 to 6/15 | 0/15 | Japan |
| Miscellaneous | 5 | B3GAT2,FOXL2, KCNK13, RAB31, and SLIT1 & FAM78A, FSTL1, KCNC1, MYODCD and SLC6A4- 2 step process | NA | NA | NA | USA, |
| Miscellaneous |  | Hierarchial Analysis | NA | NA | NA | Australia |
| Miscellaneous |  | SFRP2, IGF2 DMR0, CACNA1G, CDKN2A, MLH1, SOCS1, RUNX3, NEUROG1, LINE-1 repeat element and H19)-9520 | NA | NA | NA | UK, USA |
| Miscellaneous |  | CGH, unknown | NA | NA | NA | Spain |
| Miscellaneous | 3 & 5 | SOCS1, MINT-1 and hMLH; and NEUROG1, THBD, HAND1, ADAMTS1, and IGFBP3 | ≥2/3 in three marker panel | <2/3 group 1 but ≥3/5 group 2 methylated | <2/3 group 1 but <=3/5 group 2 methylated | United Kingdom |
|  |  |  |  |  |  |  |

Abbreviations: MINT, methylated in tumor; W-O, Weisenberger-Ogino; NA, Not available
