# Supplementary material for: Global differences in the prevalence of the CpG island methylator phenotype of colorectal cancer
Source: BMC Cancer. 2019 Oct 17;19:964. doi: 10.1186/s12885-019-6144-9 (PMC6796359; doi:10.1186/s12885-019-6144-9)
Supplement: Supplementary file 3 — Additional file 3. Funnel and Forest Plots for Meta-analysis. [file 12885_2019_6144_MOESM3_ESM.docx]

Appendix 2. Forest plots and funnel plots by methodological subtype and by continent.

Section 1: Prevalence and Differences based on Subgroup Classification

1. CIMP-H

Figure S1. Funnel plot to assess publication bias in pooled analysis of CIMP-H prevalence.

Random pooled ES | 0.22 0.21 0.24

---------------------+---------------------------------------------------

Heterogeneity chi^2 = 2548.72 (d.f. = 110) p = 0.00

I^2 (variation in ES attributable to heterogeneity) = 95.68%

Estimate of between-study variance Tau^2 = 0.01

Test of ES=0 : z= 24.61 p = 0.00

Egger's test for small-study effects:

Regress standard normal deviate of intervention

effect estimate against its standard error

Number of studies = 111 Root MSE = 4.286

------------------------------------------------------------------------------

Std_Eff | Coef. Std. Err. t P>|t| [95% Conf. Interval]

-------------+----------------------------------------------------------------

slope | -1.693632 .1184034 -14.30 0.000 -1.928304 -1.45896

bias | 1.997168 .8022317 2.49 0.014 .4071709 3.587165

------------------------------------------------------------------------------

Test of H0: no small-study effects P = 0.014

Figure S2: Forest plot of studies of CIMP-H prevalence with 95% confidence intervals. **[Au: Please define all abbreviations used on the figures in the legends, including ES and CI]**

Section II: Prevalence of Subgroups by Methodology Used to Assess CIMP

1. MINT Marker Group
2. CIMP-H

Figure S3. Funnel plot to assess publication bias in pooled analysis of CIMP-H prevalence in MINT marker group.

Random pooled ES | 0.26 0.23 0.29

---------------------+---------------------------------------------------

Heterogeneity chi^2 = 605.60 (d.f. = 37) p = 0.00

I^2 (variation in ES attributable to heterogeneity) = 93.89%

Estimate of between-study variance Tau^2 = 0.01

Test of ES=0 : z= 16.79 p = 0.00

Egger's test for small-study effects:

Regress standard normal deviate of intervention

effect estimate against its standard error

Number of studies = 38 Root MSE = 3.553

------------------------------------------------------------------------------

Std_Eff | Coef. Std. Err. t P>|t| [95% Conf. Interval]

-------------+----------------------------------------------------------------

slope | -2.129423 .1987782 -10.71 0.000 -2.532564 -1.726282

bias | 5.139148 1.279847 4.02 0.000 2.543497 7.734799

------------------------------------------------------------------------------

Test of H0: no small-study effects P = 0.000

Figure S4. Forest plot of studies of CIMP-H prevalence in MINT marker group with 95% confidence intervals.

CIMP subgroup prevalence determined by Weisenberger-Ogino panel:

Figure S5. Funnel plot to assess publication bias in pooled analysis of CIMP-H prevalence in Weisenberger-Ogino group.

Random pooled ES | 0.21 0.18 0.23

---------------------+---------------------------------------------------

Heterogeneity chi^2 = 1634.42 (d.f. = 54) p = 0.00

I^2 (variation in ES attributable to heterogeneity) = 96.70%

Estimate of between-study variance Tau^2 = 0.01

Test of ES=0 : z= 15.89 p = 0.00

. Egger's test for small-study effects:

Regress standard normal deviate of intervention

effect estimate against its standard error

Number of studies = 55 Root MSE = 5.023

------------------------------------------------------------------------------

Std_Eff | Coef. Std. Err. t P>|t| [95% Conf. Interval]

-------------+----------------------------------------------------------------

slope | -1.553382 .1716727 -9.05 0.000 -1.897714 -1.209051

bias | .8184737 1.301971 0.63 0.532 -1.792949 3.429896

------------------------------------------------------------------------------

Test of H0: no small-study effects P = 0.532

Figure S6. Forest plot of studies of CIMP-H prevalence in Weisenberger-Ogino group with 95% confidence intervals.

Method 3: Human Methylation Array

1. CIMP-H

Figure S7. Funnel plots to assess publication bias in pooled analysis of CIMP-H prevalence in human methylation array group.

Random pooled ES | 0.22 0.13 0.31

---------------------+---------------------------------------------------

Heterogeneity chi^2 = 33.54 (d.f. = 3) p = 0.00

I^2 (variation in ES attributable to heterogeneity) = 91.06%

Estimate of between-study variance Tau^2 = 0.01

Test of ES=0 : z= 4.60 p = 0.00

Egger's test for small-study effects:

Regress standard normal deviate of intervention

effect estimate against its standard error

Number of studies = 4 Root MSE = 3.786

------------------------------------------------------------------------------

Std_Eff | Coef. Std. Err. t P>|t| [95% Conf. Interval]

-------------+----------------------------------------------------------------

slope | -2.623858 1.1798 -2.22 0.156 -7.700128 2.452412

bias | 6.088025 6.275994 0.97 0.434 -20.9154 33.09145

------------------------------------------------------------------------------

Test of H0: no small-study effects P = 0.434

Figure S8. Forest plot of studies of CIMP-H prevalence in human methylation array group with 95% confidence intervals.

Section 3: Prevalence of CIMP subgroups by continents

1. Asia
2. CIMP-H

Figure S9. Funnel plot to assess publication bias in pooled analysis of CIMP-H prevalence in Asia.

Random pooled ES | 0.22 0.18 0.26

---------------------+---------------------------------------------------

Heterogeneity chi^2 = 640.70 (d.f. = 29) p = 0.00

I^2 (variation in ES attributable to heterogeneity) = 95.47%

Estimate of between-study variance Tau^2 = 0.01

Test of ES=0 : z= 11.54 p = 0.00

Egger's test for small-study effects:

Regress standard normal deviate of intervention

effect estimate against its standard error

Number of studies = 30 Root MSE = 4.319

------------------------------------------------------------------------------

Std_Eff | Coef. Std. Err. t P>|t| [95% Conf. Interval]

-------------+----------------------------------------------------------------

slope | -1.491878 .3600146 -4.14 0.000 -2.229334 -.7544213

bias | .6415611 1.957228 0.33 0.746 -3.367639 4.650762

------------------------------------------------------------------------------

Test of H0: no small-study effects P = 0.746

Figure S10. Forest plot of studies of CIMP-H prevalence in Asia with 95% confidence intervals.

1. Europe
2. CIMP-H

Figure S11. Funnel plot to assess publication bias in pooled analysis of CIMP-H prevalence in Europe.

Random pooled ES | 0.21 0.18 0.24

---------------------+---------------------------------------------------

Heterogeneity chi^2 = 690.29 (d.f. = 36) p = 0.00

I^2 (variation in ES attributable to heterogeneity) = 94.78%

Estimate of between-study variance Tau^2 = 0.01

Test of ES=0 : z= 12.41 p = 0.00

Egger's test for small-study effects:

Regress standard normal deviate of intervention

effect estimate against its standard error

Number of studies = 37 Root MSE = 3.563

------------------------------------------------------------------------------

Std_Eff | Coef. Std. Err. t P>|t| [95% Conf. Interval]

-------------+----------------------------------------------------------------

slope | -1.253432 .1933592 -6.48 0.000 -1.645972 -.8608921

bias | -.6337551 1.190364 -0.53 0.598 -3.050322 1.782812

------------------------------------------------------------------------------

Test of H0: no small-study effects P = 0.598

Figure S12. Forest plot of studies of CIMP-H prevalence in Europe with 95% confidence intervals.

1. Australia

Figure S13. Funnel plot to assess publication bias in pooled analysis of CIMP-H prevalence in Australia.

Figure S14. Forest plot of studies of CIMP-H prevalence in Australia with 95% confidence intervals.

Number of studies = 12 Root MSE = 5.767

------------------------------------------------------------------------------

Std_Eff | Coef. Std. Err. t P>|t| [95% Conf. Interval]

-------------+----------------------------------------------------------------

slope | -1.801362 .6804314 -2.65 0.024 -3.317458 -.2852664

bias | 2.106104 4.747872 0.44 0.667 -8.472814 12.68502

------------------------------------------------------------------------------

Test of H0: no small-study effects P = 0.667

1. North America
2. CIMP-H

Figure S15. Funnel plot to assess publication bias in pooled analysis of CIMP-H prevalence in North America.

Random pooled ES | 0.27 0.23 0.31

---------------------+---------------------------------------------------

Heterogeneity chi^2 = 554.81 (d.f. = 18) p = 0.00

I^2 (variation in ES attributable to heterogeneity) = 96.76%

Estimate of between-study variance Tau^2 = 0.01

Test of ES=0 : z= 12.84 p = 0.00

Number of studies = 19 Root MSE = 4.873

------------------------------------------------------------------------------

Std_Eff | Coef. Std. Err. t P>|t| [95% Conf. Interval]

-------------+----------------------------------------------------------------

slope | -2.102225 .2026327 -10.37 0.000 -2.529742 -1.674707

bias | 6.708125 1.92639 3.48 0.003 2.643797 10.77245

------------------------------------------------------------------------------

Test of H0: no small-study effects P = 0.003

Figure S16. Forest plot of studies of CIMP-H prevalence in North America with 95% confidence intervals.

1. South America
2. CIMP-H

Figure S17. Funnel plot to assess publication bias in pooled analysis of CIMP-H prevalence in South America.

Figure S18. Forest plot of studies of CIMP-0 prevalence in South America with 95% confidence intervals.

Africa:

1. CIMP-H

Figure S19. Funnel plot to assess publication bias in pooled analysis of CIMP-H prevalence in Africa

Figure S20. Forest plot of studies of CIMP-0 prevalence in Africa with 95% confidence intervals.
