# Supplementary material for: Global differences in the prevalence of the CpG island methylator phenotype of colorectal cancer
Source: BMC Cancer. 2019 Oct 17;19:964. doi: 10.1186/s12885-019-6144-9 (PMC6796359; doi:10.1186/s12885-019-6144-9)
Supplement: Supplementary file 4 — Additional file 4. Bias Assessment for Cohort Studies. [file 12885_2019_6144_MOESM4_ESM.docx]

Bias Assessment for Cohort Studies Using the New-Castle Ottawa Scale
